# Role of Single Nucleotide Polymorphism L55M in the *Paraoxonase 1* Gene as a Risk and Prognostic Factor in Acute Coronary Syndrome

**DOI:** 10.3390/cimb44120403

**Published:** 2022-11-27

**Authors:** Krastina I. Doneva-Basheva, Konstantin Gospodinov, Tanya Tacheva, Dimo Dimov, Tatyana I. Vlaykova

**Affiliations:** 1 Department of Cardiology, Medical University, 5800 Pleven, Bulgaria; 2 Department of Cardiology, Medical Faculty, Trakia University, University Hospital “Prof. Dr Stoyan Kirkovich”, 6000 Stara Zagora, Bulgaria; 3 Department of Chemistry and Biochemistry, Medical Faculty, Trakia University, 6000 Stara Zagora, Bulgaria; 4 Department of Medical Biochemistry, Medical University-Plovdiv, 4002 Plovdiv, Bulgaria

**Keywords:** PON1, genotyping, ACS, STEMI, risk

## Abstract

The aim of the current study is to explore the possible role of L55M, (rs 854560, 163T > A) SNP as a predisposing factor for acute coronary syndrome (ACS) and to assess its potency as a prognostic biomarker for short (1 year) survival and for median (5 years) and 9-year long patients’ outcome. Methods: The current work is a prospective case-control study with 77 patients with acute coronary syndrome (53 with ST-elevation myocardial infarction, STEMI, 14 with non-ST-elevation myocardial infarction, NSTEMI and 10 with unstable angina, UA) and 122 control individuals. Patients were followed-up for 9 years. The genotyping for PON1 L55M SNP was carried on by PCR-RFLP method. Results: The results of the genotyping for *PON1* L55M SNP showed a statistically significant difference (*p* = 0.023) between the controls and the whole group of patients with acute coronary syndrome, as the individuals with genotype with at least one variant M allele had about 2.5-fold higher risk for developing ACS than those which are homozygous of the wild-type L allele (LL genotype). In patients with variant M allele genotypes (LM + MM) which suffer from non-ST-segment elevation ACS (NSTEACS, i.e., UA or NSTEMI), the serum levels of total cholesterol (TC) and triacylglycerols (TAG) are significantly higher than in NSTEACS patients with LL genotype (*p* = 0.022 for TC and *p* = 0.015 for TAG). There was no significant difference in the survival rate at the 1st, 5th and 9th year of follow-up between ACS patients with different genotypes, although it is worth to note that in the subgroup of NSTEACS, all patients (*n* = 13) with variant M allele genotypes (LM + MM) were alive at the end of the first year, while 2 of the patients with LL genotype (18.2%) were dead. Conclusions: The results of our current study suggest that the variant M allele and the M allele genotypes (LM + MM) of the PON1 L55M polymorphism are risk factors for acute coronary syndrome, especially for patients with STEMI, but do not support the possible effect of this polymorphism on the clinical progression and outcome of the patients with ACS either in short or long follow-up periods.

## 1. Introduction

Coronary heart disease (CHD) is the leading cause of morbidity and mortality in both developed and developing countries [1]. Important risk factors for that multifunctional disease are the low-density lipoproteins (LDL) and the glycation and oxidative modification of LDL, which take main role in atherogenesis [2,3]. The high-density lipoproteins (HDL) are independent protective factors against atherosclerosis, which is the pathogenic basis of coronary heart disease. HDL complexes have been found to possess high antioxidative and antiglycative properties, mainly due to the enzyme activity of paraoxonase 1 (PON1) [3,4].

Although many factors play a role in atherogenesis, the low PON1 activity has been shown to be an independent risk factor [2,5]. Paraoxonase was initially associated with its hydrolytic activity against organic phosphate compounds [3,4]. The name of the enzyme is based on its ability to hydrolyse the organic phosphate substrate paraoxon (paraoxonase activity EU 3.1.8.1), which is a toxic metabolite of the insecticide parathion [6].

PON1 belongs to a family of paraoxonases consisting of three isoenzymes: PON1, PON2 and PON3. The three genes encoding these enzymes are localized in tandem on the long arm of chromosome 7 (7q21.3-q22.1) [7]. PON1 and PON3 are expressed in liver and secreted into the bloodstream where they are associated with HDL [3,4]. PON2 is not present in blood but is expressed in multiple tissues, including liver, lung, brain and myocardium [6]. PON1 is a glycoprotein with Ca^2+^ dependent hydrolase activity (paraoxonase/arylesterase), which is able to decompose esters mainly of acetic acid (phenylacetate, tio-phenylacetate, 2-naphtylacetate) and toxic oxonium metabolites (paraoxon, diasoxon) of organic phosphate insecticides and nerve-paralyzing agents (zoman, sarin) [8,9]. Since those compounds are non-physiological, the paraoxonase/arylesterase activity of PON1 is clearly not the physiological function of the enzyme. The main natural biological activity of PON1 was proven to be the lactonase activity. The enzyme hydrolyzes a variety of aromatic and aliphatic lactones, including the lactones of hydroxyl derivatives of polyunsaturated fatty acids (PUFAs). The enzyme is found also to catalyze the reaction of lactonization of γ–and δ –hydroxy carboxylic acids and to hydrolyse also the cyclic carbonates and some phospholipid oxidation products, such as isoprostane, carboxyl and aldehyde esters and hydroperoxides of phosphatidylcholine, thus possessing a phospholipase A2-like activity [8,9,10].

Therefore, it is assumed that the lactones, hydroxyl and oxidation derivatives of polyunsaturated fatty acids (PUFAs) and other lipids probably are the major endogenous substrates of the enzyme [11].

PON1 is an enzyme present in several alloenzyme (variant) forms due to polymorphisms in the coding region of the gene. Until now there are described at least two single-nucleotide polymorphisms (SNPs) in coding sequences with functional activity: Gln(Q)/Arg(R) substitution at position 192 and Leu (PON1 Q192R rs662, 575 A > G) and (L)/Met(M) substitution in position 55 (PON1 L55M, rs 854560, 163 T > A) [6,12]. The Q192R influences the substrate specificity of the hydrolytic activity of PON, while the L55M SNP affects the concentration of the enzyme in the serum, being higher in the LL genotype than MM genotype [12]. The L allele has been associated with higher PON1 mRNA. The effect of L55M SNP, however, is thought to be due to linkage disequilibrium to some promoter polymorphism affecting the gene transcription, rather than to the direct effect of the nucleotide substitution [13].

There are large amount of data clearly demonstrating the role of decreased PON1 enzyme activity in coronary artery disease (CAD) [14], however the association studies for the role of the two functional polymorphisms in PON1 gene (PON1L55M, and Q192R) with ischemic heart disease (CHD) and acute myocardial infarction risk are still with controversial results [15,16,17,18,19,20,21].

So far, there are a very limited number of studies concerning the effects of PON1 polymorphisms on the severity and progression of hearth diseases [22].

In this respect the aim of the current study is to explore the possible role of PON1L55M, (rs 854560, 163T > A) SNP as a predisposing factor for acute coronary syndrome and to assess its potency as a prognostic biomarker for short (1 year) survival and for median (5 years) and 9-year long patients’ outcome.

## 2. Materials and Methods

### 2.1. Patients and Control Individuals

In the current study, 77 patients with acute coronary syndrome and 122 control individuals were recruited. The demographic and clinical characteristics of the patients and controls are presented in Table 1.

Patients were recruited randomly, when they were hospitalized with acute coronary syndrome in the Department of Internal Medicine, Medical Faculty, University Hospital, Trakia University, Stara Zagora, Bulgaria (UMBAL-Stara Zagora) and in the Specialized Hospital for Hearth Diseases, Yambol, Bulgaria (SBALK-Yambol), during the period of January 2009–February 2010. The patients were followed-up for a period of more than 9 years by regular examinations and telephone checking.

All patients were involved in the study according to several inclusion and exclusion criteria: Inclusion criteria: age above 18 years, criteria fulfilling the diagnostic algorithm for STEMI, NSTEMI, UA according to European guidelines [23]. Exclusion criteria: age below 18 years.

The control group has been selected according to the case-control method during preventive examinations among employees of the Faculty of Medicine, after signed informed consent by the participants. All control subjects have been surveyed for comorbidities, and those with a history of ischemic disease have been excluded.

All patients and control individuals have given their approval for their survey following and have written an informed consent. The protocol of the current study was issued by the Ethics committee at Medical Faculty, Trakia University, Stara Zagora, Bulgaria (No3/21/04/2008 and No16/19.03.21).

### 2.2. Clinical Examination and Standard Laboratory Analyses

During the hospitalization of all patients, blood was taken to examine blood count, blood sugar, total cholesterol, LDL-cholesterol, HDL-cholesterol, triglycerides, creatinine, creatine phosphokinase (CPK), CPK-MB, troponin I (Tn I), which are part of the mandatory diagnostic algorithm. Most of the laboratory tests (myocardial necrosis enzymes are mandatory) are followed in dynamics, part of the algorithm for diagnosis and treatment of ACS. Elevation of enzymes for myocardial necrosis is accepted for the following values: CPK ≥ 195 U/L, CPK-MB ≥ 25 U/L, troponin I ≥ 0.1 ng/mL for those examined in UMBAL-Stara Zagora and SBALK-Yambol.

Electrocardiography- The patient’s ECG has been recorded on a Schiller electrocardiograph device at baseline and then repeatedly during follow-up. A standard 12-lead ECG has been used with a paper speed of 25 mm/s and gain of 10 mm/mV. If necessary, the right thoracic and lateral leads have been additionally recorded. The localization of AMI and ST-T changes has been determined from the electrocardiogram.

Echocardiography-On the day of hospitalization, until the 2nd hour for AMI with/without ST-elevation and up to the 12th hour for UA, an Echocardiography examination has been carried out using a Phillips IE 33 device in the intensive sector of the cardiology department of the UMBAL-Stara Zagora and SBALK-Yambol. A standard echocardiographic protocol has been used for each patient. The examination has been performed in the left lateral position. The standard positions have been used: parasternal position long and short axis, apical position in four-cavity, two-cavity and five-cavity sections. Two-dimensional imaging (2D), M mode, Color Doppler, Pulse Wave Doppler, Continuous Wave Doppler, and Tissue Doppler have been used. Ejection fraction (EF) has been measured by Simpson’s method. Segmental kinetics (normokinesia, hypokinesia, akinesia and dyskinesia) have been evaluated at each of the described slices in a 16-segment model. Transthoracic echocardiography includes-left ventricular ejection fraction.

Global Registry of Acute Coronary Events (GRACE) score, which is important for predicting in-hospital and 6 months mortality of patients with ACS was calculated [24] for all patients and the risk has been assessed based on the accepted classification [25].

### 2.3. Isolation of Genomic DNA

Genomic DNA was isolated from the whole venous blood using a commercial kit, based on the column chromatography (GenElute™ Mammalian Genomic DNA Miniprep Kit, Sigma, Burlington, MA, USA). The isolation was performed from 200 µL venous blood according to the manual of the kit. The purity and the quality of the isolated genomic DNA were assessed spectrophotometrically as described earlier [26].

### 2.4. PCR-RFLP Method of Genotyping for PON1 L55M (rs 854560)

The genotyping for PON1L55M (rs 854560) was performed by applying a PCR-RFLP method, described by Suehiro et al. [27]. In brief: Two to 4 μL genomic DNA (about 40–50 ng) were used as a template for the PCR reactions. The amplification mixes with a final volume of 30 μL contained 1xPCR buffer, 1 pmol/μL of each of the primers (Table 2), 100μM dNTP, 1.5 mM MgCl_2_, a 1 U Tag polymerase (STS DNA polymerase, STS Ltd., Sofia, Bulgaria).

There were performed 35 cycles of amplification reaction with annealing temperature of 61 °C. After the last cycle, a final extension of 10 min was done at 72 °C. 

The restriction reaction was performed for 16 h at 37 °C within a final volume mix of 25 μL, containing 20 μL of PCR product, 5U *NlaIII* and 1xBuffer G (ThermoFisherScientific Ltd., Waltham, MA USA).

### 2.5. Agarose Gel Electrophoresis

To prove the quality of the PCR reaction and for separation of the products after the restriction reactions used for genotyping, a 3% agarose gel electrophoresis was performed. The agarose gels were prepared in 1x TBE buffer (90 mM Tris-boric acid, 1 mM Na_2_EDTA, pH8). The electrophoresis was run in equipment for submarine electrophoresis (Clever Scientific Ltd., Rugby, UK), for 30–45 min under currency of 5–6 V/cm distance between the electrodes. The results were visualized with ethidium bromide on UV transilluminator and were documented with gel documentation system (Syngene, Synoptics Ltd., UK).

**Figure 1:**
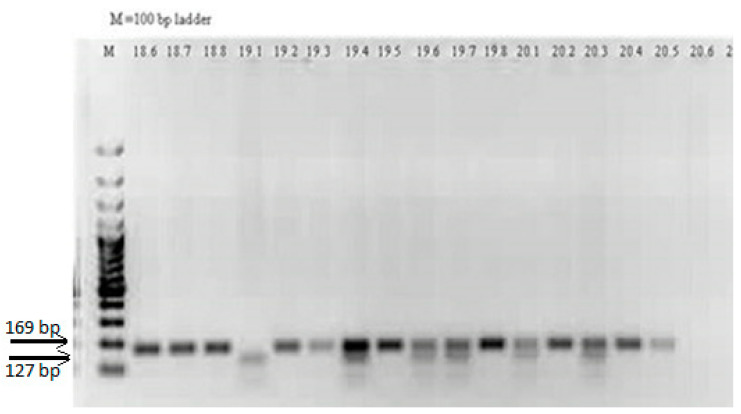
Agarose gel electrophoresis of the restriction products obtained in PCR-RFLP genotyping analysis for *PON1* L55M (163T > A, rs 854560) SNP.

The PCR fragment had a length of 169 bp. The SNP in the variant allele introduces a restriction site for *NlaIII*, which leads to the production of two fragments of 127 bp and 42 bp. Because the 42 bp fragment is too small, in the electrophoresis it was invisible. Thus, individuals homozygous with the wild L allele (LL) had in the electrophoresis only one band of 169 bp, heterozygous individuals (LM) had two bands of 169 bp and of 127 bp, and those which are homozygous with the variant M allele (MM) had one band of 127 bp. (Figure 1).

LL genotype–lines 18.6, 18.7, 18.8, 19.2, 19.3, 19.5, 19.8, 20.2, 20.4, and 20.5; LM genotype–lines 19.4, 19.6, 19.7, 20.1 and 20.3; MM genotype-line 19.1; NTC–line 20.6; M–100 bp ladder.

### 2.6. Statistical Analyses

Statistical analyses were performed using SPSS 16.0 for Windows (IBM, Chicago, IL, USA). The continuous variables were compared either by Student *t*-test or One-way ANOVA test with LSD Post hoc analysis, or by using Mann-Whitney U test or Kruskal-Wallis H test depending on the normality of the distribution. The correlations between the continuous variables were assessed using the Spearman correlation test.

The frequency differences between the categorical data in the groups, including the genotypes and alleles, were assessed with χ^2^ test in 2 × 2 or 2 × 3 contingency tables. The odds ratios (OR) and 95% confidence interval (CI) were obtained by applying the binary Logistic regression. 

The Kaplan-Mayer method was applied for drawing the cumulative survival curves and the difference in the survival was calculated with Log rank test. The prognostic significance of various factors regarding patients’ survival in different follow-up periods were assessed with the univariate and multivariate Cox regression analyses. Factors with *p* < 0.05 were considered statistically significant.

## 3. Results

### 3.1. PON1 L55M SNP and the Risk for ACS

The results of the genotyping for *PON1* L55M (163T > A, rs 854560) SNP showed a statistically significant difference (*p* = 0.023) between the controls and the whole group of patients with acute coronary syndrome (Table 3). Twenty-eight (36.4%) of the patients had LL genotype, 39 (50.6%) were heterozygous (LM), and 10 patients (13.0%) were homozygous for the variant M allele (MM genotype). The genotype distribution of the control group was as follows: 69 (56.6%) were with LL genotype, 42 (34.4%) with LM genotype and 11 with MM genotype (Table 3). This genotype distribution determines more than 2-fold higher risk of developing acute coronary syndrome when an individual is a carrier of genotypes with the variant M allele of *PON1* L55M (Table 3). The statistical significance remained also after adjustment for sex and age (Table 3).

When the genotype distribution was analyzed in the groups of patients with different diagnosis and was compared with the distribution in the controls, we found that significant difference existed only between the patients with STEMI and controls (*p* = 0.012) (Table 4), while there was no difference in the genotype distribution between the controls and patients with UA (*p* = 0.433) and NSTEMI (*p* = 0.819) (Table 4). 

So, individuals having a genotype with at least one variant M allele of the *PON1* L55M (163T > A, rs 854560) SNP appears to have more than 2.5-fold higher risk for developing STEMI than those which are homozygous of the wild-type L allele (LL genotype) (Table 4).

### 3.2. Associations of PON1 L55M SNP with Biochemical and Clinical Markers of Patients with ACS

**Figure 2:**
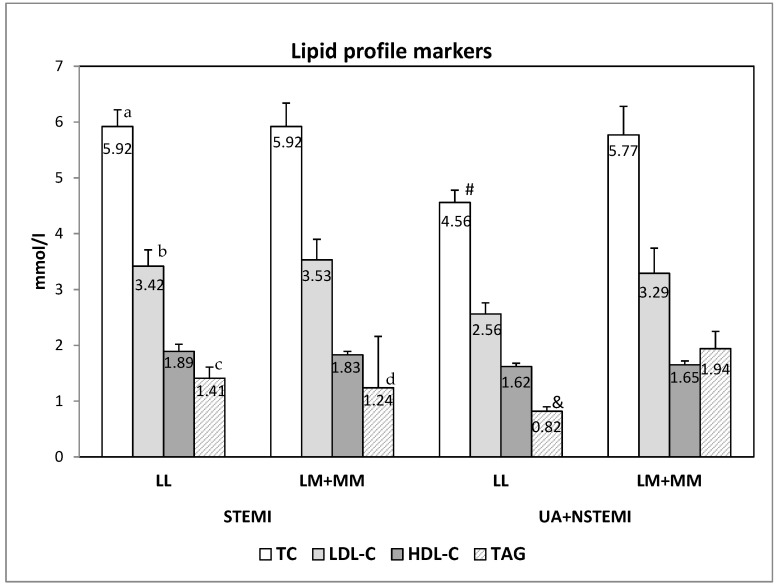
The serum lipid profile markers in patients with different PON1 L55M genotypes and diagnosis. The data are presented as mean ± SEM (standard error of mean). (a,b and c—differences between the STEMI and NSTEACS (UA + NSTEMI) with LL genotype; d—difference between the STEMI and NSTEACS (UA + NSTEMI) with LM + MM genotype; # and &—difference between LL and LM + MM genotypes of patients with NSTEACS).

The analyses of the association of the genotypes with the lipid profile serum characteristics and serum markers of kidney functions (creatinine and glomerular fraction), showed that in patients with genotype with variant M allele (LM + MM) which suffer from non-ST-segment elevation ACS (NSTEACS, i.e., UA or NSTEMI), the serum levels of total cholesterol (TC) and triacylglycerols (TAG) are significantly higher than in NSTEACS patients with LL genotype (*p* = 0.022 for TC and *p* = 0.015 for TAG) (Figure 2, Table 5).

When the lipid profile markers and the markers for renal function were compared between patients with different diagnosis but with the same genotype, we observed significantly higher levels of total cholesterol (*p* = 0.005), LDL-cholesterol (*p* = 0.042) and TAG (*p* = 0.029/0.053) in patients with LL genotype with STEMI than the patients with LL genotype with NSTEACS (Figure 2, Table 5). In the patients with M allele genotypes (LM + MM), the only difference was seen for TAG, being significantly higher in NSTEACS patients than with STEMI (*p* = 0.034) (Figure 2, Table 5).

Based on the criteria for assessing dyslipidemia (TC > 5.5 mmol/L; LDL-C > 3.5 mmol/L; HDL-C < 1.29 mmol/L in women and < 1.03 mmol/L in men; TAG > 1.7 mmol/L), the same group of patients (patients with NSTEACS having LM or MM genotype) were significantly more frequently (76.9%, 10 of 13) with dyslipidemia than the patients with LL genotype, suffering from the same disease (UA or NSTEMI, 36.4%, 4/11, *p* = 0.045). On the other hand, but without significance, the dyslipidemia was more frequently in patients with LL genotype suffering from STEMI (70.6%, 12/17) than those with LM or MM genotypes with STEMI (58.3%, 21/36, *p* = 0.390). 

When comparing the markers for kidney function (Table 5), we found a tendency (*p* = 0.072) without statistical significance, for higher serum creatinine in patients with M allele genotypes (LM or MM genotype) who are with NSTEACS than patients with the same diagnosis, but with LL genotype. When the patients were classified into groups according to the upper limit of normal ranges of 134 µmol/L creatinine, the tendency remained: patients with M allele genotypes (LM or MM genotype) who are with NSTEACS were more frequently with higher creatinine (23.1%, 3/13) than the patients with the same diagnosis but with LL genotype, who all had normal creatinine values (*p* = 0.089). No difference was seen for the GFR in patients with different diagnosis and different genotypes (Table 5). 

We did not obtain any prevalence of the presence of risk factors such as arterial hypertension (*p* = 0.955), diabetes mellitus (*p* = 0.847), overweight/obesity (*p* = 0.723), smoking (current/ex-smokers, *p* = 0.914), familial predisposition (*p* = 0.141) between patients with different *PON1 L55M* genotypes.

### 3.3. PON1 L55M SNP and other Factors Influencing the Survival of the Patients with ACS

Patients were followed-up in the periods of 12 months (1 year), 60 months (5 years) and 108 months (9 years) after the admission to the hospitals because of the incidence of acute coronary disease. After 1 year follow-up, patients who survived were 60 (survival rate of 77.9%): 38 out of 53 with STEMI (71.7%) and 22 out of 24 (91.7%) of the patients with NSTEACS (UA + NSTEMI, *p* = 0.050) (Table 6).

No associations were obtained between the 1-year survival rate and PON1 L55M genotypes neither in the whole patients’ group (*p* = 0.640), nor in the subgroups with different diagnosis (*p* = 0.902 for STEMI; *p* = 0.108for UA + NSTEMI). However, it is worth noting that in the subgroup of NSTEACS, all patients (*n* = 13) with variant M allele genotypes (LM + MM) were alive at the end of the first year, while 2 of the patients with LL genotype (18.2%) were dead.

After 5 years of follow-up, the patients who were alive were altogether 49 (63.6% survival rate). No associations were observed with the PON1 SNP (*p* = 0.561). The same lack of association of the PON1 SNP (*p* = 0.389) was found after 9 years follow-up of the patients, when the survival rate was 50.6% (39 out of 77).

No association was also found between the short-term risk of mortality (in-hospital mortality, GRACE score ≤108/109–139/≥ 140) with the genotypes of *PON1 L55M* SNP (*p* = 0.436). No significant associations were also observed between the genotypes distribution and the long-term risk of mortality (GRACE score ≤88/89–117/≥ 118, *p* = 0.302).

The Kaplan-Maier survival curve after one year of following of the patients shows that there was no significant difference in the survival of the patients with LL genotype (mean survival period of 17.81 months) and those patients with M allele genotypes (LM + MM, mean survival period of 20.31 months, *p* = 0.553, Log rank test) (Figure S1A-supplementary). A lack of significant difference between carriers of different genotypes was obtained both in the subgroup of patients with STEMI (Figure S1B-supplementary), and with NSTEACS (*p* = 0.115) (Figure S1C-supplementary).

In the middle period of 5 years (60 months) follow-up, the visible difference between the survival of the patients with LL and those with M allele genotypes (LM_MM) is completely lost (Figure S2A-supplementary). Even in the long period of 9 years (108 months) follow-up, although not significantly, the prognosis of the patients with LL genotype becomes more favorable than that of the patients with LM or MM genotypes (Figure S2B-supplementary), especially for the patients with NSTEACS (Figure S2C-supplementary).

In Cox’s univariate analysis, several demographics, clinical and biochemical parameters had statistically significant adverse effects on the short-, middle- and long period survival of patients with ACS. For the short period survival (1 year) the main factors which determined the unfavorable outcome are the age ≥60 years (*p* = 0.003), GFR below 60 (ml/min/1.73m^2^), previous incident of myocardial infarction with ST-segment elevation (*p* = 0.113 for whole group, *p* = 0.038 for STEMI), experience of cardiogenic shock (*p* = 0.002), lung edema (*p* = 0.026), cardiac asthma (*p* = 0.008), ST-segment elevation (*p* = 0.025), cardiac arrest (*p* = 0.007), EF < 50 (*p* = 0.011), Triple-vessel disease (TVD) (*p* = 0.031), Atrioventricular (AV) block (*p* = 0.001), CAD > 50 (*p* = 0.131 for the whole group, *p* = 0.056 for STEMI) (Table 7).

In the COX’s multivariate analysis when all above-mentioned factors including the PON1 L55M genotypes were included, it has appeared that only the presence of cardiogenic shock (*p* = 0.015) and of the AV block (*p* = 0.018) remained significant independent prognostic factors (Table 7); TVD had a marginal significance as an unfavorable independent factor (*p* = 0.056), while all other factors have lost their role (Table 8).

We also performed the COX’s multivariate analyses for the survival periods of 5 years and 9 years, including the same factors as those for the one-year survival period (Table 8). The results showed that for the 5 years period again the presence of cardiogenic shock (*p* = 0.013), the AV block (*p* = 0.039) and TVD (*p* = 0.027) were the significant independent prognostic factors (Table 8). For the long follow-up period of 9 years, the independent unfavorable factors were the incidence of early infarction (*p* = 0.006), the presence of cardiac shock (*p* = 0.040) and TVD (*p* = 0.006).

## 4. Discussion

The Acute coronary syndrome (ACS), as it is described by The American Heart Association (AHA), is a complex of three coronary artery disease (CAD): ST segment elevation myocardial infarction (STEMI), non-ST segment elevation myocardial infarction (NSTEMI) or unstable angina (UA). The main common feature of them is the sudden block of the blood supplied to the heart muscle [28].

There a variety of risk factors for ACS, some non-modifiable (age, gender, ethnicity, and genetics) and other modifiable, such as high blood pressure, high cholesterols, especially LDL cholesterol, high fasting plasma glucose, diabetes, physical inactivity, overweight or obesity, ambient and household air pollution, and smoking [29,30].

Because the increased LDL levels are considered one of the strong risk factors, the proper metabolism and the protection of LDL from oxidative and/or glycation modification are in the focus of the research aiming to find out new targets for therapy and to discriminate individuals with higher probability for developing of CAD including those of ACS in order for early and proper prophylaxis [30]. 

Among the factors which are involved in protection of LDL from oxidative and glycation modification is the PON1 enzyme, carried by HDL and contributing to the HDL antioxidant properties by hydrolyzing the oxidative derivatives of lipids, including those in LDL [4,31,32].

It is already proven that the serum levels and the enzyme activity and substrate specificity of PON1 are influenced by gene variants, one of which is PON1 L55M SNP. The study of Garin et al., has provided a strong support for the marked modulation of plasma concentrations of PON1 by L55M SNP as the MM genotype is associated with significantly lower levels [12,33].

This type of functional analyses has given a strong rationality for evaluation of possible role of polymorphisms in the gene of PON1 as factors modulating the risk for developing and progression of diseases associated with oxLDL.

The emerging numbers of reports with results from cross-sectional association studies, the relationship between PON1 L55M polymorphism and different cardiovascular diseases have produced inconsistent results.

In several studies, the variant allele or genotype of PON1 L55M polymorphism are predictors of CVD, but in other there was no significant difference in genotype frequencies between patients and controls and those studies failed to find an association with coronary heart disease (CHD) and acute myocardial infarction [16,19,34].

The first large meta-analysis for different types of CHD includes 21 studies based which gave rise to 23 separate comparisons: 9 of them have involved a total of 3189 cases of myocardial infarction (MI) and 3650 controls, while the other 14 have involved a total of 2800 cases of coronary stenosis and 4466 controls. The results of this meta-analysis have not shown significant association of CHD with the L55M polymorphism [17]. Later, another larger meta-analysis consisting of 64 studies including over 19,000 patients and 33,397 healthy individuals has been performed to examine the association between the two PON1 polymorphisms (Q191R and L55M) and cardiovascular disease (CVD). It has established that L55M shows a significant association with heart disease, as the 55MM genotype determined 1.44-fold higher risk in Europeans (OR 1.44, 95% CI 1.34–1.56) and 1.21-fold higher risk in Asians (OR 1.21, 95% CI 1.04–1.42) [20].

There are also reports with opposite findings: the 55M allele, as well as the 55MM genotype, appeared to be with a protective role for coronary artery disease (CAD) in comparison to the wild type L allele [21].

In our current study, we evaluated the role of PON L55M polymorphism in acute coronary syndrome (ACS) and found a statistically significant difference in genotype frequency between patients and controls. The heterozygous genotype (LM), as well as the genotypes with variant M allele (LM + MM) appeared to determine more than 2.5-fold higher risk for developing of ACS, which was independent of the confounding factors as age and gender. More specifically, the PON1 55MG genotype and genotypes with variant M allele were predisposing factors for STEMI, while for UA or NSTEMI, these associations were not significant. Our results are in accordance with those of Bounafaa et al., which also have shown that the PON1 55MM genotype is associated with a higher risk for ACS in comparison to the individuals with LL genotype (OR = 3.69; 95% CI = 1.61–11.80) [31].

When comparing a variety of biochemical markers between the L55M PON1 genotypes, we found relations only in the group of patients with NSTEACS: those patients with genotypes with at least one variant M allele (LM + MM) had significantly higher serum levels of total cholesterol (TC) and triacylglycerols (TAG) than the patients with LL genotype. The observed difference in the levels of TC and TAG between the carriers of different genotypes could be explained with the functional effect of the variant PON1 M55 allele, being associated with lower enzyme concentration than 55L allele which further might lead to lower defense role against hypercholesterolemia and trigliceridemia. However, these associations were not seen in patients with STEMI, as well as in the whole patients’ group with ACS. Similar lack of differences between the carriers of PON1 L55M genotypes was reported for patients with CAD [21] and ACS [31].

In our study, we paid a lot of attention to the possible relations of PON1 L55M genotypes with the standard risk factors, with the clinical characteristics and with clinical outcome of the patients with ACS. However, we did not find any statistically significant associations of the genotypes with survival rate at the 1st, 5th and 9th year of follow-up, and most of the standard risk factors. We have only observed that all patients with NSTEACS with variant M allele genotypes (LM + MM) were alive at the end of the first year, while 2 of the patients with LL genotype (18.2%) died. Log-rank test and Univariate Cox’s analysis did not describe significant differences in any of the follow-up periods. Thus, we can conclude that the L55M polymorphism has no remarkable impact on the progression and outcome of the patients with ACS. These results are in line with the recent once reported by Huang et al., 2022 [22], which have not also found any associations of PON1 L55M, rs 854560with the severity of ischemic stroke (IS) in the Chinese population. However, they have described relation of promoter −909G > C polymorphism (rs85457) in PON1 with the severity of the stroke as the GG genotype was associated with a mild stroke [22], Grzegorzewska et al., has explored the three single nucleotide variants of PON1 in relation with cardiovascular mortality in conventional cigarette smokers and non-smokers treated with hemodialysis [35]. Concerning the PON1 L55M SNP (T > A, rs854560) they have obtained that the T allele (L allele) is associated with lower cardiovascular mortality in non-diabetic smokers, while the TT (LL) genotype expresses a negative, although non-significant, correlation with non-CHD-related cardiac death in non-smokers. 

In our study, we also focused on exploring the markers and factors that impact on the survival of patients with ACS. For the short 1-year survival, it has appeared that the main unfavorable are above 60 years, GFR below 60 mL/min/1.73m^2^, earlier incident of myocardial infarction with ST-segment elevation, experience of cardiogenic shock, lung edema, cardiac asthma, ST-segment elevation, cardiac arrest, EF < 50, TVD and AV block. Among them, independent remain to be only the presence of cardiac shock and the AV block. All these markers are previously described in a vast number of publications as prognostic factors for survival of patients with heart diseases, which proves their usefulness in the clinical practice [36,37,38].

We are aware that our study has several limitations that should be considered taken into account when assessing the value of the obtained results. The most important limitation is that the study cohorts are relatively small, especially those of UA and NSTEMI. The second is that the control group does not much completely on age and gender to the patients groups. To avoid this limitation in interpreting the results, we have applied the multivariate Logistic regression analysis with adjustment to age and sex. Another limitation is that we did not present PON1 paraoxonase and arylesterase activity of patients and controls, because we were able to assess the enzyme activity only for small part of the cohorts. The reason for this incompleteness is that fresh serum/plasma is required for enzyme activity assessment, but such was not available for all patients and controls. That is why a larger study which includes more patients with ACS and with analyzed PON1 enzyme activity and/or protein concentration is warranted in order to confirm our current observations and conclusions.

## 5. Conclusions

The results of our current study suggest that the variant M allele and the M allele genotypes (LM + MM) of the PON1 L55M polymorphism are risk factors for acute coronary syndrome, especially for patients with STEMI. The possible explanation could be the functional effect of the variant M allele on the expression level of the gene, leading to a decreased concentration of the enzyme in the serum of the individuals with those genotypes and further for lowering the protective HDL antioxidant activity. However, our results, although showing some associations with biochemical parameters, do not support the possible effect of this polymorphism on the clinical progression and outcome of the patients with ACS either in short or long follow-up periods.

## Figures and Tables

**Table 1:** Demographic and clinical characteristics of the patients with acute coronary syndrome and of the control individuals.

Indicators	Patients N (%)	Controls N (%)	*p*-Value
	77	122	
Sex			0.006
Males	49 (63.6)	53 (43.4)	
Females	28 (36.4)	69 (56.6)	
Age (years)			<0.0001
Mean ± SEM	64.53 ± 1.36	56.99 ± 1.17	
BMI (kg/m^2^)			0.618
Mean ± SEM	27.13 ± 0.52	26.81 ± 0.39	
BMI	(77)	(101)	0.504
Normal	21 (27.3)	34 (33.7)	
Overweight	41 (53.2)	45 (44.6)	
Obese	15 (19.5)	22 (21.8)	
Smoking habits	(77)	(111)	0.636
Non-smokers	46 (59.7)	72 (64.9)	
Ex-smoker	6 (7.8)	10 (9.0)	
Current smokers	25 (32.5)	29 926.1)	
Total cholesterol (TC) (mmol/L) Mean ± SEM	5.70 ± 0.23	5.02 ± 0.47	0.220
HDL-cholesterol (mmol/L) Mean ± SEM	1.78 ± 0.04	1.86 ± 0.23	0.934
LDL-cholesterol (mmol/L) Mean ± SEM	3.32 ± 0.20	2.42 ± 0.46	0.071
Triglycerides (TG) (mmol/L)Mean ± SEM	1.33 ± 0.11	1.14 ± 0.22	0.507
Creatinine (µmol/L) Mean ± SEM	100.56 ± 3.53	NA	
GFR (mL/min/1.73 m^2^) Mean ± SEM	69.06 ± 2.63	NA	
Troponin (ng/mL) Mean ± SEM	13.63 ± 10.64	NA	
CPK (U/L) Mean ± SEM	665.26 ± 111.16	NA	
CPK-MB (U/L) Mean ± SEM	93.09 ± 15.44	NA	
LVEF	(77)	NA	
≤29%	5 (6.5)		
30–39%	2 (2.6)		
40–49%	29 (37.7)		
≥50%	41 (53.2)		
Diabetes	(77)	NA	
No	53 (68.8)		
D2T	15 (19.5)		
D1T	6 (7.8)		
Glucose intolerance	3 (3.9)		
Diagnosis	(77)		
STEMI	53 (68.8)		
UA	10 (13.0)		
NSTEMI	14 (18.2)		
Blood vessels affected	(74)	NA	
none	6 (8.1)		
one vessel	26 (35.1)		
two vessels	15 (20.3)		
three vessels	27 (36.5)		
GRACE score		NA	
Mean ± SEM	120.95 ± 3.86		
GRACE score–short period risk		NA	
Low risk (≤108)	26 (33.8)		
Moderate risk (109–139)	31 (40.2)		
High risk ≥140	20 (26.0)		
GRACE score–long period risk		NA	
Low risk (≤88)	12 (15.6)		
Moderate risk (89–117)	26 (33.8)		
High risk (≥118)	39 (50.6)		
1–year survival			
Alive	57 (74.0)		
Dead	20 (26.0)		
5–year survival			
Alive	49 (63.3)		
Dead	28 (36.4)		
9–year survival			
Alive	39 (50.6)		
Dead	38 (49.4)		

NA—not available/not applicable; CPK—Creatine phosphokinase; CPK-MB—MB (heart) fraction of Creatine phosphokinase; LVEF—Left ventricular ejection fraction; BMI—body mass index.

**Table 2:** Primer sequences, size of the PCR product and restriction fragments, the annealing temperature and restriction enzyme used in PCR-RFLP method for genotyping for *PON1 L55M* (rs 854560).

Primers	Annealing Temperature	Restrictase	PCRand Allele-Specific Products
L55MF (5′- GAA GAG TGA TGT ATA GCC CCA G-3′) L55MR (5′- ACT CAC AGA GCT AAT GAA AGC CA-3′),	55 °C	NlaIII (37 °C)	PCR product–169 bp L allele–169 bp M allele–127 + 42 bp (not visible).

**Table 3:** Genotype and allelic distribution of L55M in PON1 in patients with acute coronary syndrome and controls.

L55M SNP in PON1	Patients		Controls		OR (95% CI), *p*-Value	OR (95% CI), *p*-Value
	N	Frequency	N	Frequency		(After Adjustment for Sex and Age)
	n = 77	(%)	n = 122	(%)		
Genotype distribution						
LL	28	36.4	69	56.6	1.0 (referent)	
LM	39	50.6	42	34.4	**2.288 (1.233–4.248), *p* = 0.009**	**2.390 (1.230–4.643), *p* = 0.010**
MM	10	13	11	9	2.240 (0.856–5.865), *p* = 0.100	2.275 (0.973–7.782), *p* = 0.056
LM + MM	49	63.6	53	43.4	**2.278 (1.268–4.095), *p* = 0.006**	**2.457 (1.306–4.622), *p* = 0.005**
Allele distribution						
55L	104	62.7	180	73.8	1.0 (referent)	
55M	62	37.3	64	26.2	**1.677 (1.098–2.561), *p* = 0.017**	

**Table 4:** Genotype and allelic distribution of L55M in PON1 in patients with different diagnosis and controls.

	Patients		Controls		OR (95% CI), *p*-Value	OR (95% CI), *p*-Value (After Adjustment for Sex and Age)
	N	Frequency	N	Frequency		
		(%)		(%)		
**Genotype distribution–patients with STEMI**						
	*n* = 53		*n* = 122			
LL	17	32.1	69	56.6	1.0 (referent)	
LM	29	54.7	42	34.4	**2.803 (1.376–5.706), *p* = 0.005**	**2.750(1.304–5.798), *p* = 0.008**
MM	7	13.2	11	9	2.583(0.872–7.652), *p* = 0.087	3.029 (0.950–9.658), *p* = 0.061
LM + MM	36	67.9	53	43.4	**2.757 (1.398–5.436), *p* = 0.003**	**2.801(1.372–5.718), *p* = 0.005**
**Genotype distribution–patients with UA**						
	*n* = 10	(%)	*n* = 122	(%)		
LL	4	40	69	56.6	1.0 (referent)	
LM	4	40	42	34.4	1.643 (0.390–6.920), *p* = 0.499	1.834(0.419–8.029), *p* = 0.421
MM	2	20	11	9	3.136 (0.512–19.22), *p* = 0.216	4.967 (0.713–34.63), *p* = 0.106
LM + MM	6	60	53	43.4	1.953 (0.542–7.273), *p* = 0.318	2.295(0.590–8.927), *p* = 0.231
**Genotype distribution–patients with NSTEMI**						
	*n* = 14	(%)	*n* = 122	(%)		
LL	7	50	69	56.6	1.0 (referent)	
LM	6	42.9	42	34.4	1.408 (0.443–4.474), *p* = 0.562	1.597 (0.462–5.527), *p* = 0.460
MM	1	7.1	11	9	0.896(0.100–8.005), *p* = 0.922	2.301 (0.214–24.80), *p* = 0.492
LM + MM	7	50	53	43.4	1.302(0.430–3.939), *p* = 0.640	1.670 (0.505–5.523), *p* = 0.400

**Table 5:** The serum lipid profile markers and the markers for renal function in patients with different PON1 L55M genotypes and diagnosis. The data are presented as mean ± SEM (standard error of mean).

Indicators	STEMI			LL	NSTEACS (UA + NSTEMI)			LM + MM
	LL (*n* = 17)	LL_LM (*n* = 36)	*p*-Value (LL vs. LM + MM)	*p*-Value– (STEMI vs. UA + NSTEMI)	LL (*n* = 11)	LL_LM (*n* = 13)	*p*-Value (LL vs. LM + MM)	*p*-Value– (STEMI vs. UA + NSTEMI)
TC (mmol/L) Mean ± SEM	5.92 ± 0.30	5.92 ± 0.43	0.112	**0.005**	4.56 ± 0.22	5.77 ± 0.51	**0.022**	0.919
LDL-C (mmol/L) Mean ± SEM	3.42 ± 0.29	3.53 ± 0.37	0.265	**0.042**	2.56 ± 0.21	3.29 ± 0.45	0.106	0.768
HDL-C (mmol/L) Mean ± SEM	1.89 ± 0.13	1.83 ± 0.06	0.864	0.208	1.62 ± 0.06	1.65 ± 0.08	0.955	0.113
TAG (mmol/L) Mean ± SEM	1.41 ± 0.20	1.24 ± 0.16	0.299	**0.029**	0.82 ± 0.08	1.94 ± 0.31	**0.015**	**0.034**
Creatinine (µmol/L) Mean ± SEM	100.24 ± 5.54	102.54 ± 5.40	0.614	0.578	87.36 ± 6.61	106.63 ± 8.05	*0.072*	0.511
GFR (ml/min/1.73 m^2^) Mean ± SEM	64 ± 5.54	69.97 ± 4.05	0.620	0.134	77.10 ± 6.80	65.50 ± 5.70	0.361	0.483

**Table 6:** Survival rate after one, 5 and 9 years of follow-up of patients with ACS.

Survival Periods	STEMI N (%)	NSTEACS N (%)	*p* Value (STEMI vs. NSTEACS)
1 year survival Alive Dead	38 (71.7) 15 (28.3)	22 (91.7) 2 (8.3)	**0.050**
5-year survival			
Alive Dead	32 (60.4) 21 (39.6)	17 (70.8) 7 (29.2)	0.377
9-year survival			
Alive Dead	26 (49.1) 27 (50.9)	13 (54.2) 11 (45.8)	0.678

**Table 7:** Univariate Cox’s proportional analysis of the short period survival (one year) of patients with acute coronary syndrome.

	The Whole Group with ACS			STEMI			NSTEACS		
Factor (N)	*p*-Value	HR	CI (95%)	*p*-Value	HR	CI (95%)	*p*-Value	HR	CI (95%)
Age	**0.003**			**0.006**			0.440		
<60 years		**1**			**1**			1	
≥60 years		**4.82**	**1.70–13.70**		**4.57**	**1.56–13.43**		103.73	0.001–1351
GFR	0.027			0.202			0.456		
≥60 mL/min/1.73m^2^		**1**			1			1	
≤60 mL/min/1.73m^2^		**3.07**	**1.14–8.32**		1.96	0.70–5.51		304.56	0.000–1036
Previous incidence of infarction	0.161	1 1.98	0.76–5.12	**0.020**	**1** **3.34**	**1.21–9.26**	0.832	1 0.74	0.05–11.86
No									
Yes									
Cardiogenic shock	**0.002**			**0.006**			NA		
No		**1**			**1**				
Yes		**7.73**	**2.17–27.56**		**6.17**	**1.70–22.35**			
Lung edema	**0.026**			**0.016**			0.884		
No		**1**			**1**			1	
Yes		**4.15**	**1.19–14.53**		**4.80**	**1.34–17.29**		0.047	0.000–8242
Cardiac asthma	**0.008**			**0.043**			NA		
No		**1**			**1**				
Yes		**3.91**	**1.42–10.79**		**2.99**	**1.04–8.65**			
ST-segment elevation	**0.025**			0.335			NA		
No		**1**			1				
Yes		**5.41**	**1.24–23.66**		24.99	0.04–1724			
Cardiac arrest	**0.007**			**0.002**			0.884		
No		**1**			**1**			1	
Yes		**5.58**	**1.59–19.63**		**7.94**	**2.18–28.91**		0.047	0.000–8242
LVEF	**0.011**			**0.030**			0.545		
≥50		**1**			**1**			1	
<50		**4.32**	**1.41–13.26**		**4.08**	**1.15–14.49**		2.36	0.15–37.68
Affected blood vessels 1 or 2 3 (TVD)	**0.031**	**1** **2.89**	**1.10–7.61**	**0.007**	**1** **4.19**	**1.48–11.80**	0.992	1 1.15	0.07–18.38
AV block	**0.001**			**0.005**			NA		
No		1			1				
Yes		8.07	2.25–28.93		6.49	1.78–23.69			
CAD	0.131			***0.056***			0.658		
<50		1			***1***			1	
≥50		2.37	0.77–7.27		***3.43***	***0.97–12.17***		0.54	0.03–8.56
PON1 L55M SNP	0.555		0.51–3.52	0.789		0.40–3.39	0.451	82.72 1	0.001–8050
LL		1.34			1.16				
LM + MM		1			1				

N—number of cases; LN—lymph node metastases; HR—hazard ratio; CI—confidence interval, NA—not assessable; GFR—Glomerular filtration rate; Atrioventricular (AV) block; CAD—coronary artery disease; TVD—Triple-vessel disease; LVEF—Left ventricular ejection fraction.

**Table 8:** Multivariate Cox’s proportional analysis for 1-year, 5-year and 9-year survival periods of patients with acute coronary syndrome.

	1 Year’s Period			5 Years’ Period			9 Years’ Period		
Factor (N)	*p*-Value	HR	CI (95%)	*p*-Value	HR	CI (95%)	*p*-Value	HR	CI (95%)
Age	***0.093***			0.237			0.117		
<60 years		***1***			1			1	
≥60 years		***3.60***	***0.81–16.09***		1.18	0.68–4.86		1.97	0.84–4.60
GFR	0.776			0.310			0.750		
≥60 mL/min/1.73m2		1			1			1	
≤60 mL/min/1.73m2		0.78	0.77–4.75		2.24	0.47–10.67		0.85	0.31–2.35
Previous incidence of infarction No Yes	0.528	1 1.60	0.37–6.95	***0.092***	1 2.24	0.88–5.72	**0.006**	**1** **3.15**	**1.39–7.14**
Cardiogenic shock	**0.015**			**0.013**			**0.040**		
No		**1**			**1**			**1**	
Yes		**62.80**	**2.20–1790**		**16.31**	**1.81–146.81**		**8.10**	**1.10–59.61**
Lung edema	0.265			0.412			0.685		
No		1			1			1	
Yes		3.50	0.39–31.84		1.86	0.42–8.18		1.34	0.33–5.53
Cardiac asthma	0.146			0.132			0.646		
No		1			1			1	
Yes		4.11	0.61–27.77		2.74	0.74–10.14		1.33	0.40–4.41
ST-segment elevation	0.296			0.450			0.135		
No		1			1			1	
Yes		2.96	0.39–22.72		1.58	0.48–5.19		2.26	0.78–6.54
Cardiac arrest	0.291			0.297			0.580		
No		1			1			1	
Yes		3.85	0.31–50.00		2.56	0.44–14.71		1.52	0.34–6.80
LVEF	0.424			0.261			0.240		
≥50		1			1			1	
<50		1.81	0.42–7.69		1.77	0.65–7.77		1.66	0.71–3.82
Affected blood vessels	***0.056***			**0.027**			**0.009**		
1 or 2		***1***			**1**			**1**	
3 (TVD)		***6.90***	***0.96–49.79***		**4.61**	**1.19–17.87**		**4.91**	**1.49–16.20**
AV block	**0.018**			**0.039**			0.209		
No		**1**			**1**			1	
Yes		**14.53**	**1.58–133.59**		**6.11**	**1.09–34.14**		2.79	0.57–13.66
CAD	0.149			0.230			0.226		
<50		1			1			1	
≥50		5.82	0.53–63.50		2.54	0.55–11.68		2.20	0.62–7.86
PON1 L55M SNP	0.310		0.55–6.25	0.916		0.41–2.72	0.958		0.46–2.28
LL		1.87			1.05			1.02	
LM + MM		1			1			1	

N—number of cases; LN—lymph node metastases; HR—hazard ratio; CI—confidence interval; NA–not assessable; GFR—Glomerular filtration rate; AV-block—atrioventricular (AV) block; CAD—coronary artery disease; TVD—Triple-vessel disease; LVEF—Left ventricular ejection fraction.

## Data Availability

The date of this study is available as SPSS file when requested to the authors.

## Supplementary Materials

The following supporting information can be downloaded at: https://www.mdpi.com/article/10.3390/cimb44120403/s1, Figure S1: The Kaplan Mayer survival curves of patients with different PON1 L55M genotypes and diagnosis after 1 year of follow-up period. 3A—the whole group with ACS; 3B—patients with STEMI; 3C—patients with NSTEACS. Figure S2: The Kaplan Mayer survival curves for 5 year (4A) and 9 year (4B) follow-up of the patients from the whole group with ACS but with different PON1 L55M genotypes. 4C—The Kaplan Mayer survival curves for 9 year follow-up of the patients with NSTEACS but with different PON1 L55M genotypes.
