# Supplementary material for: Role of Single Nucleotide Polymorphism L55M in the Paraoxonase 1 Gene as a Risk and Prognostic Factor in Acute Coronary Syndrome
Source: Curr Issues Mol Biol. 2022 Nov 27;44(12):5915–32. doi: 10.3390/cimb44120403 (PMC9776864; doi:10.3390/cimb44120403)
Supplement: Supplementary file 1 [file cimb-44-00403-s001.zip › cimb-1941282-supplementary.pdf]

Figure S1. The Kaplan Mayer survival curves of patients with different PON1 L55M genotypes and diagnosis after 1 year of follow-up period. 3A- the whole group with ACS; 3B-patients with STEMI; 3C – patients with NSTEACS.

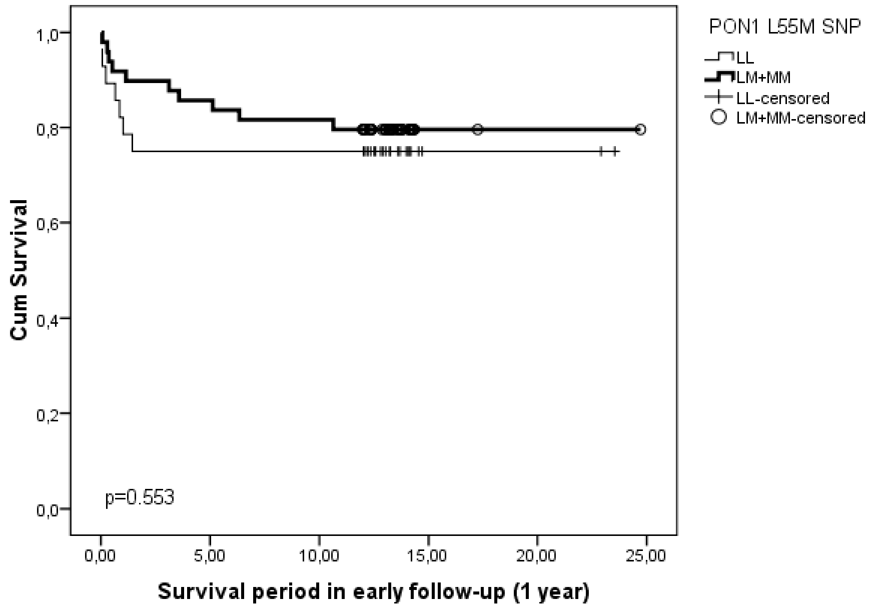

Figure S1. A.

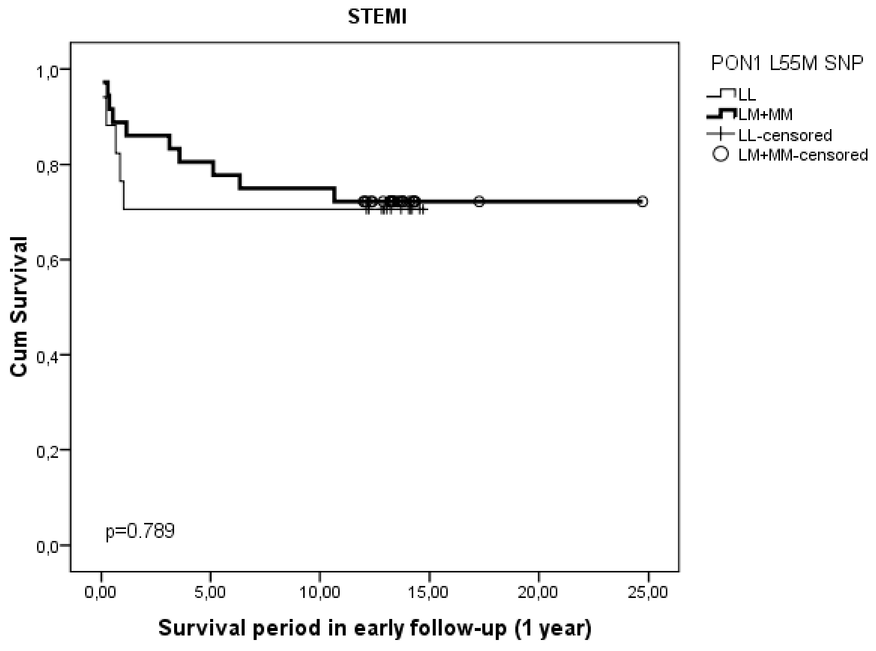

Figure S1. B.

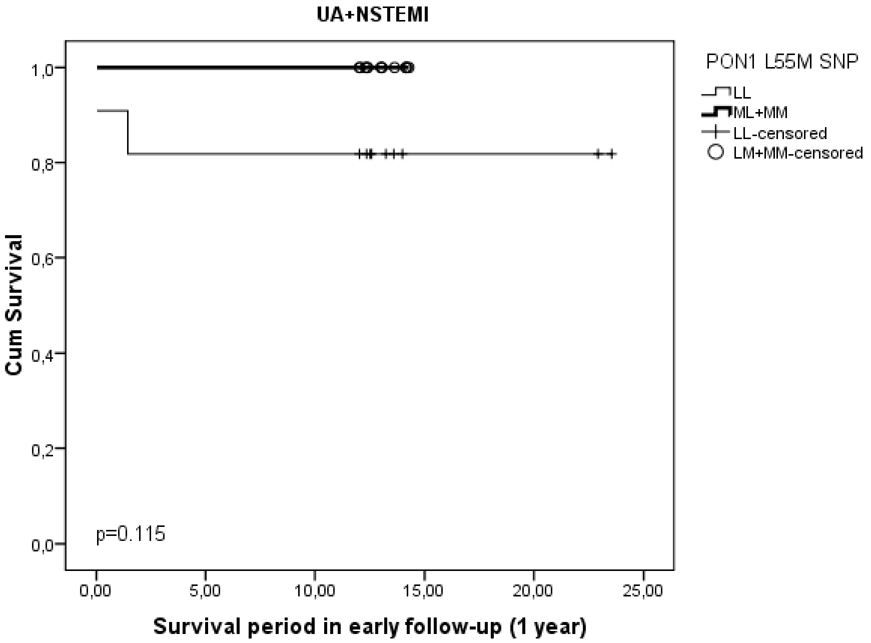

Figure S1. C.

Figure S2. The Kaplan Mayer survival curves for 5 year (4A) and 9 year (4B) follow-up of the patients from the whole group with ACS but with different PON1 L55M genotypes. 4C- The Kaplan Mayer survival curves for 9 year follow-up of the patients with NSTEACS but with different PON1 L55M genotypes.

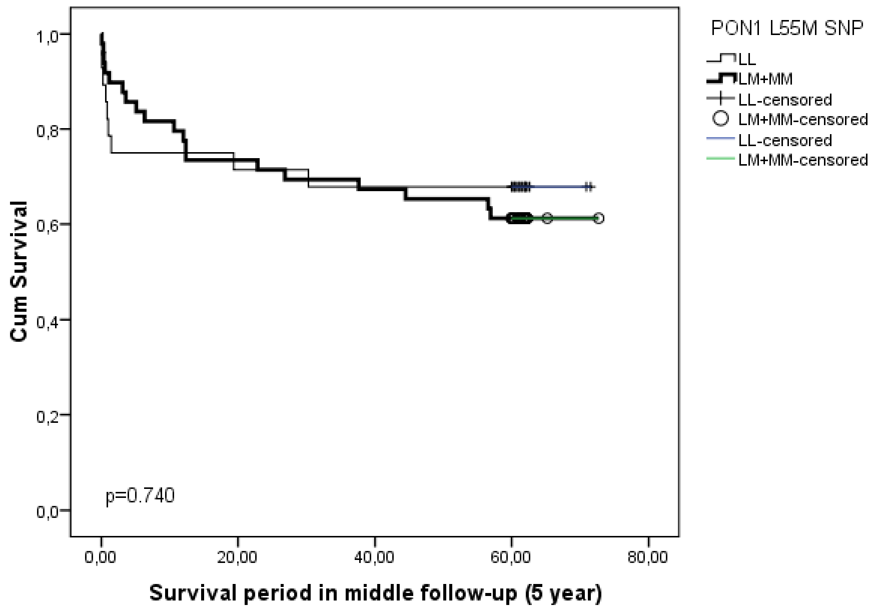

Figure S2. A.

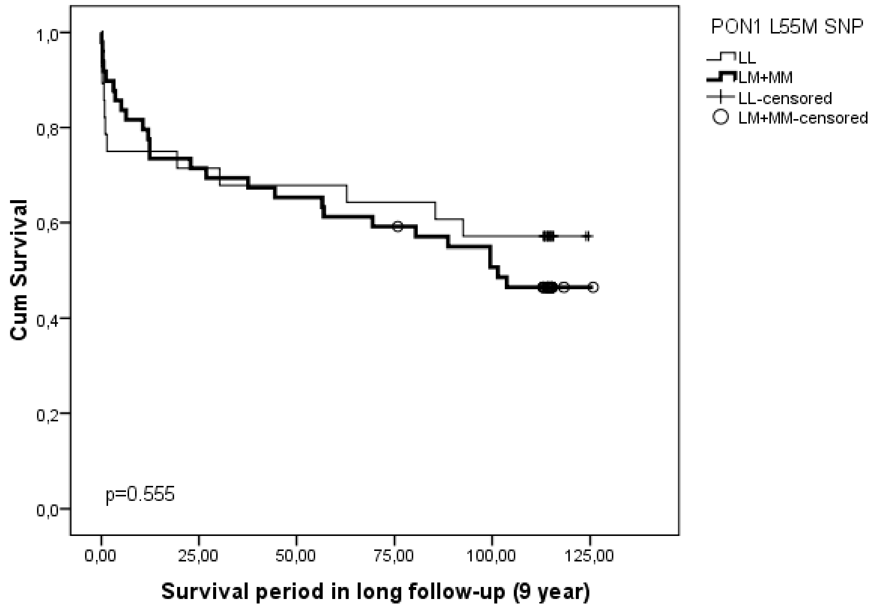

Figure S2. B.

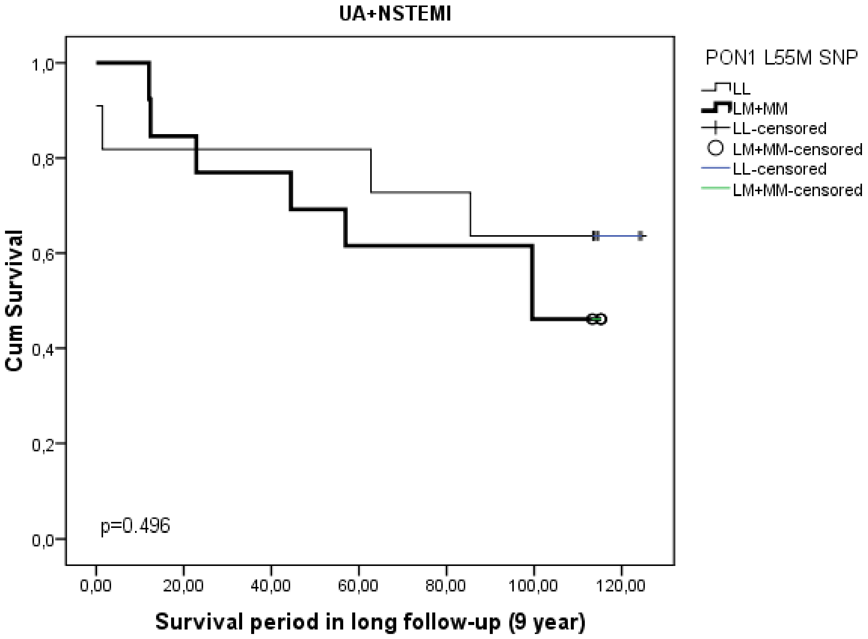
UA+NSTEMI
PON1 L55M SNP
LL
LM+MM
LL-censored
LM+MM-censored
LL-censored
LM+MM-censored
Cum Survival
1,0
0,8
0,6
0,4
0,2
0,0
p=0.496
0,00
20,00
40,00
60,00
80,00
100,00
120,00
Survival period in long follow-up (9 year)

Figure S2. C.
